# Oral tissues regeneration using intraoral mesenchymal stem cells

**DOI:** 10.4317/jced.56810

**Published:** 2021-03-01

**Authors:** Pascale Fagalde, David Reininger

**Affiliations:** 1 DDS, private practice; 2 DDS, PhD, Master in Oral Surgery and Implantology, Assistant professor, Universidad Mayor, Chile

## Abstract

**Background:**

Oral pathologies or some treatments can cause facial and functional alterations, being fundament to retrieve those functions restoring the original anatomy of the lost tissues. On this purpose, various techniques have been studied, one of these was the tissue engineering. Mesenchymal stem cells (MSC) are multipotent adult stem cells. The MSC in the oral cavity have been striking for regenerative therapies by its high plasticity, good interaction with scaffolds and growth factors, good proliferation and differentiation, they are also easy to obtain. Objective: The objective of this study was to describe the current uses of the intraoral MSC for the regeneration of the tissues of the oral cavity.

**Material and Methods:**

An electronic research was made in the databases PubMed, Cochrane Library, Google Scholar, Scopus and EBSCO between 2000 to 2018.

**Results:**

21 articles were included. 13 were studies in vivo and 8 were studies in humans. The site mostly used as a giver site was the dental pulp. Intraoral MSC are able to regenerate the pulp dentin complex, alveolar bone and periodontium.

**Conclusions:**

Intraoral MSC come from easy access areas, less traumatic interventions and have high potential to regenerate intraoral tissues in comparison to MSC from other sites of the body which allows a more predictable oral tissues regeneration.

** Key words:**Oral stem cells, oral cavity, regeneration, tissue engineering.

## Introduction

Mesenchymal stem cells are multipotent adult stem cells. They were discovered by Friedenstein and his collaborators in the 70s, who conducted studies to determine the biological characteristics of mesenchymal stem cells derived from the bone marrow (1). One of its main functions is to maintain and repair cells in the tissue in which they are found, as well as maintain the cell population. Among its most important characteristics is that they have the ability to differentiate into adipocytes, chondrocytes and osteoblasts in *in vitro* conditions. In addition, mesenchymal stem cells have the ability to evade the immune system by being immunomodulatory, which allows them to be used with therapeutic roles (1,2). 

The first site intervened to obtain mesenchymal stem cells was the bone marrow of adult patients. Although its characteristics were optimal, it was observed that the number of progenitor cells in adult tissue was quite low compared to the total number of cells extracted, in a ratio of 1 / 104-106. This is why it was necessary to carry out *in vitro* expansions to increase their number. In addition, the number of cells decreased as a function of increasing the patient’s age (3). On the other hand, obtaining mesenchymal stem cells from the bone marrow proved to be a very invasive, painful procedure with infectious complications (1).This is why they started looking for new sites that had mesenchymal stem cells which would allow a minimum of discomfort for the patient and that were present in greater quantities (1,4).

From this, it is that the oral cavity became one of the most accessible sites for obtaining mesenchymal stem cells (1), in which different sites have been identified that possess them such as the bone marrow of the alveolar bone (BMSCs), the oral mucosa (OMSCs), the periosteum (PSCs), the salivary glands (SGSCs), the adipose tissue (ASC), the dental pulp (DPSCs), the dental pulp of exfoliated teeth (SHEDs), the periodontal ligament (PLSCs), the dental follicle (DFSCs), the dental germ (GDSCs), the apical papilla (SCAP) and the inflamed periapical tissues (iPAPs) (5).

Existing reviews describe mostly *in vitro* studies, with bone regeneration being the most performed action. Therefore, the aim of this review is to extend the search of the different regenerative uses that intra-oral mesenchymal stem cells present not only at the level of bone regeneration, but also to describe their uses in other types of tissues of the oral cavity, including exclusively studies *in vivo* or in humans.

## Material and Methods

A review of the literature was carried out between 2000 and 2018 in the databases PubMed, Cochrane Library, EBSCO, Scopus and Google Scholar, performing the search strategy detailed in Table 1.

**Table 1:**
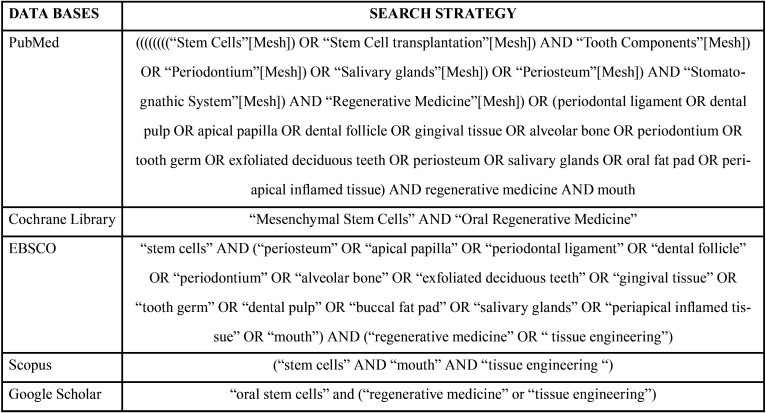
Search strategy according to database.

All studies carried out *in vivo* and in humans, prospective and retrospective cohort, clinical trials, case/control studies, including articles in English, Spanish and French, where intraoral mesenchymal stem cells were used (regardless of the autologous intraoral donor site) to regenerate intraoral defects were included. We excluded all those *in vitro* studies, studies that did not specify the mesenchymal stem cell used, studies that included patients or animals with some underlying disease or that were under pharmacological therapy that affected the regeneration of tissues, immunocompromised or immunosuppressed, patients that had been irradiated in the craniofacial region in the last 6 months or were in treatment with intravenous bisphosphonates and in the case of orally bisphosphonates should not be more than 3 years.

## Results

**Figure 1:**
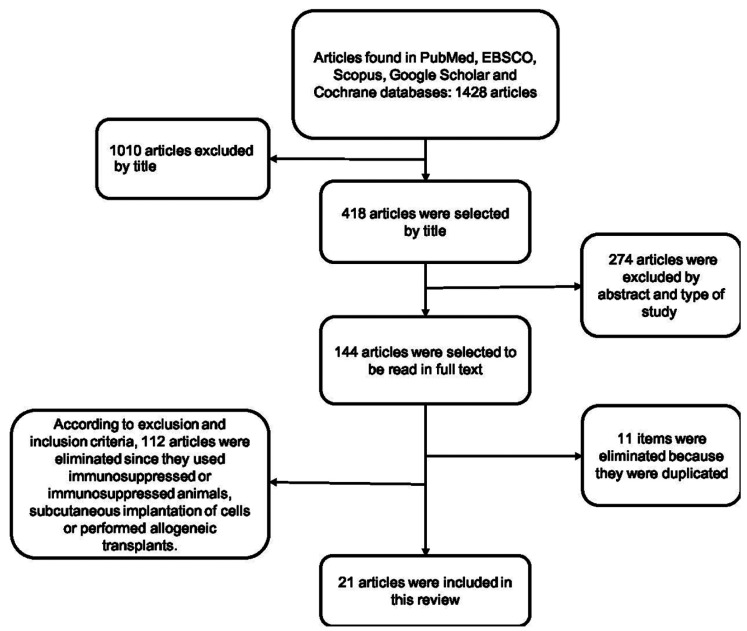
Summary of search results.

**Figure 2:**
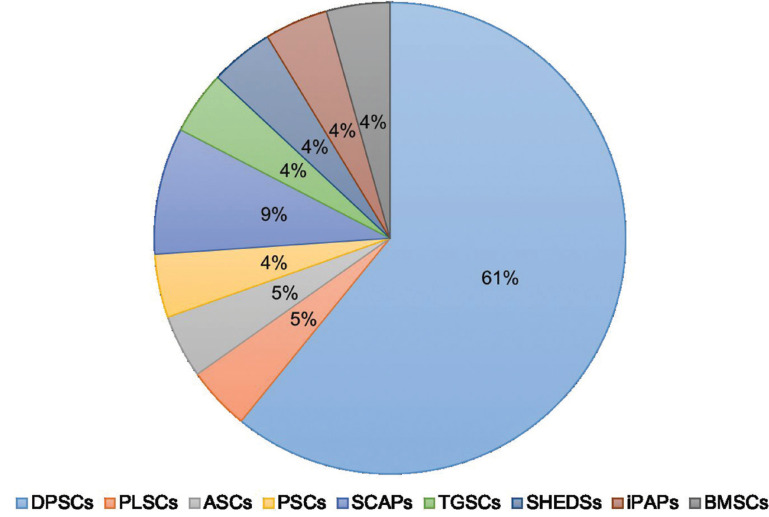
Types of cells used in the included studies.

**Figure 3:**
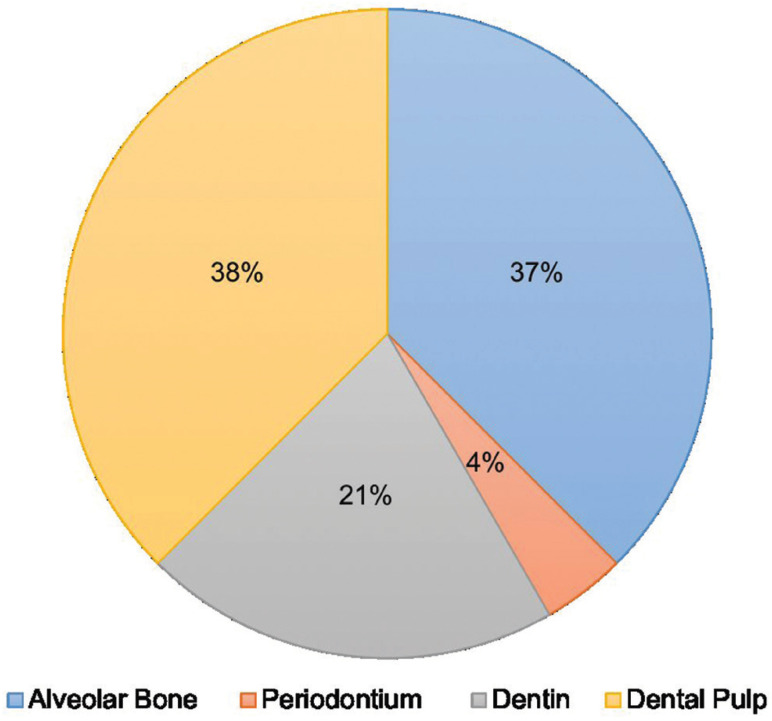
Regeneration objective of the included studies.

From the electronic search, a total of 1428 publications were found, selecting a total of 21 articles according to the inclusion and exclusion criteria (Fig. 1, Table 2,  Table 2 cont.,  Table 2 cont.-1,  Table 2 cont.-2). Of the 21 articles included, 13 (61.9%) corresponded *in vivo* studies and 8 (38,1%) corresponded humans studies. In relation to the mesenchymal stem cells, the mesenchymal stem cell of the dental pulp was the most used in a total of 14 articles (60.8%), followed by the cells of the apical papilla used in 2 articles (9.5%) (Fig. 2). In relation to the scaffolds the most used was atelocollagen, used in 5 (23.8%) articles followed by PRP (platelet rich plasma) used in 3 (14.2%) articles. 38% of the included articles had as main objective the use of mesenchymal cells is pulp regeneration, followed by alveolar bone regeneration with 37% (Fig. 3).

**Table 2:**
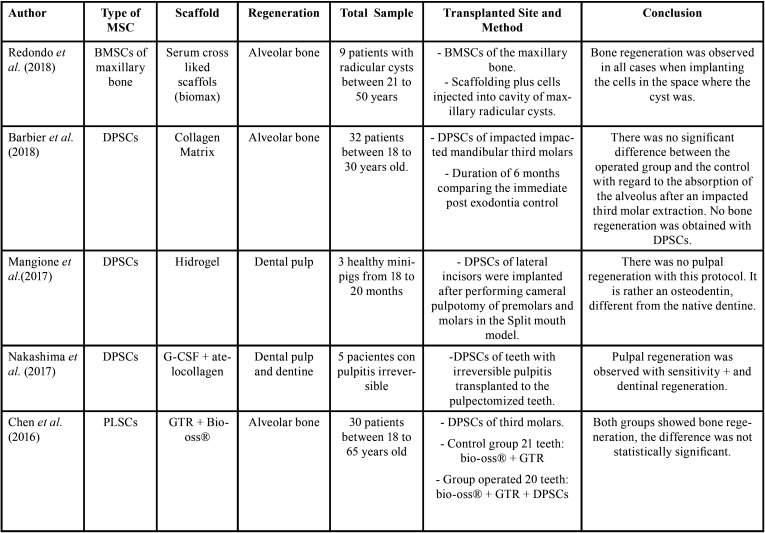
Summary of the included studies.

**Table 2 cont.**
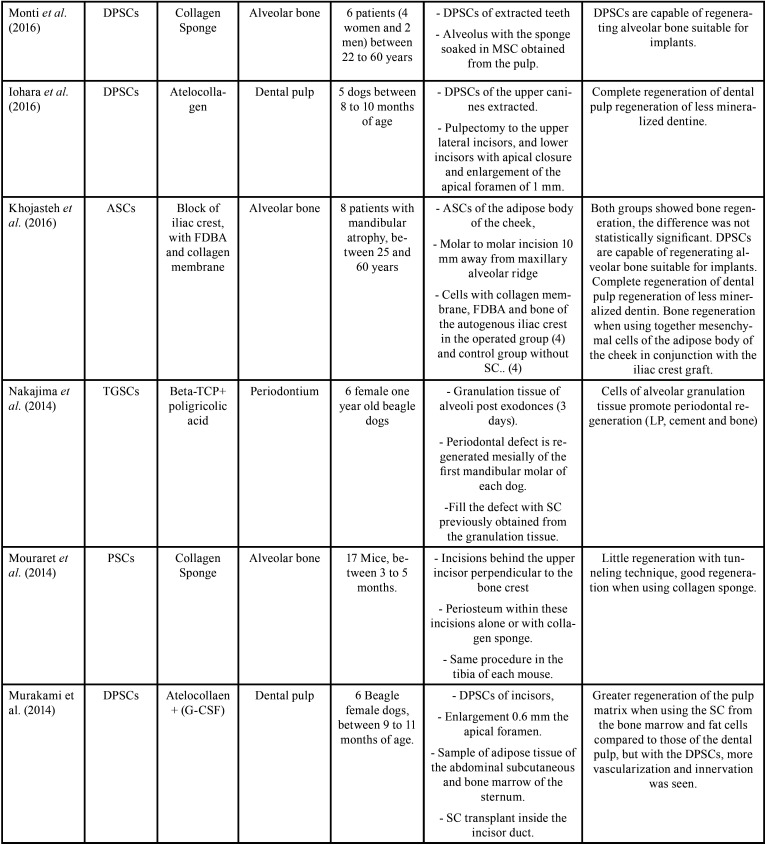
Summary of the included studies.

**Table 2 cont.-1:**
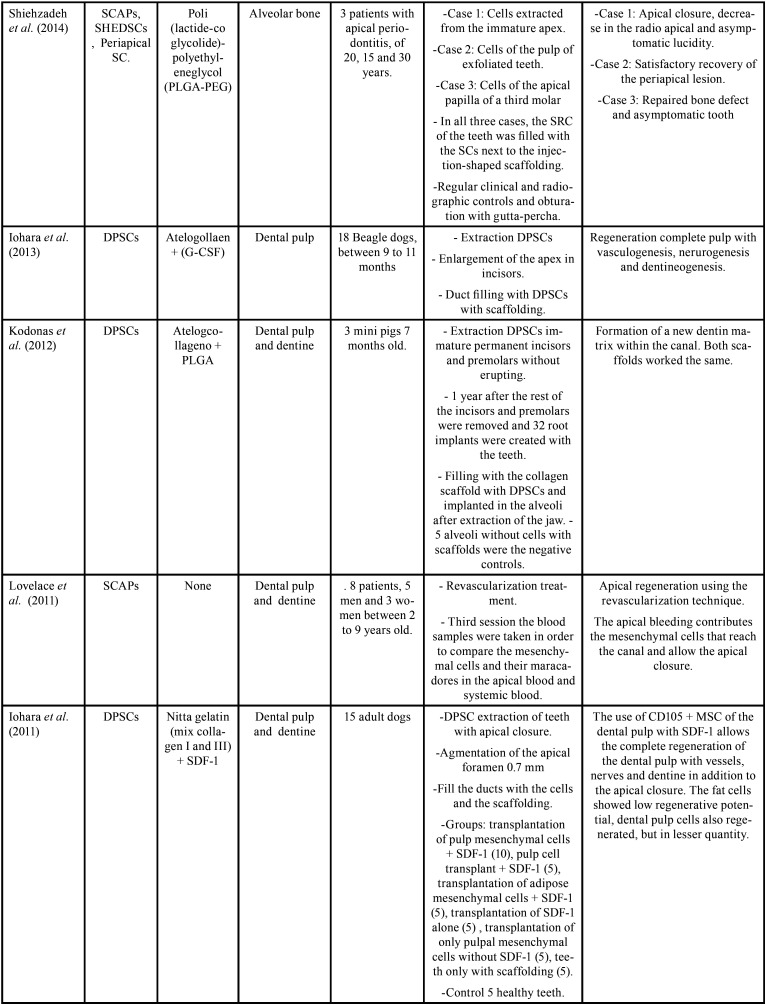
Summary of the included studies.

**Table 2 cont.-2:**
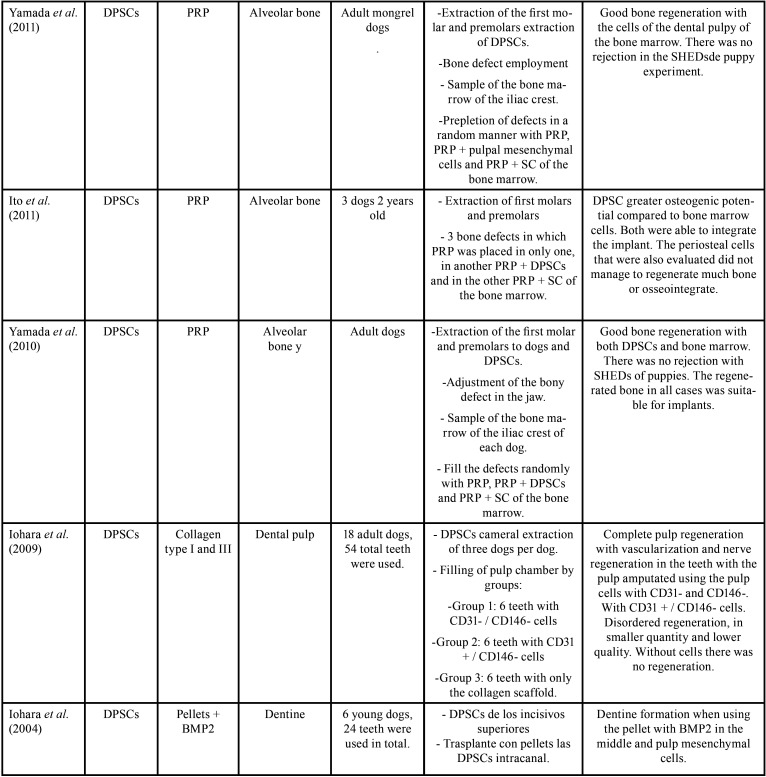
Summary of the included studies.

## Discussion

Clinical application and regeneration of oral tissues

-Regeneration of the dentin-pulp complex

38% of the included studies studied pulp regeneration, and 21% dentin. Of the studies that evaluated the pulpal regeneration, in 44.4% they also evaluated the dentinal regeneration; therefore, it is seen that there is a relation between pulp and dentine regeneration.

Of the *in vivo* studies, all used MSCs from the dental pulp. Iohara *et al.* in 2009 (6) conducted a clinical trial where they used mesenchymal stem cells of the dental pulp (DPSCs) of dogs which were isolated by flow cytometry which allowed them to obtain cells with surface markers CD31 - / CD31+ and CD146- which would correspond to a sub fraction of these DPSCs. The results obtained by a histological analysis showed the neoformation of vascular and neural tissue, as well as dental pulp inside the teeth that obtained cell transplantation CD31- / C146- and CD31+ / CD146-. But better and greater regeneration was obtained when using cells with the surface markers CD31- / C146-.

Other studies have seen the potential of CD105+ surface markers found in DPSCS. Iohara *et al.* in 2011 (7) conducted a study where they managed to isolate this cellular sub fraction by using Stromal Cell Derived Factor 1 (SDF-1). Complete pulp regeneration in dog’s teeth was also observed by histological analysis.

According to the previously studies, pulp regeneration seems to be more effective when using mesenchymal stem cells with CD31- or CD105+ compared to those where they only use pulp mesenchymal stem cells without determining the sub fraction (8,9).

Other cell isolation techniques have been further investigated since the safety of cells with CD31- and CD105+ surface markers isolated by flow cytometry has not been established and the use of SDF-1 has not been approved for clinical use (9). This is why Murakami *et al.* in 2015 (9) used granulocyte colony stimulating factor (G-CSF) to induce mobilization with the aim of isolating the sub fractions of the mesenchymal pulp stem cells. The results obtained cells with a cell phenotype similar to cells that have CD105 + with high angiogenic and neurogenic potential(9). Iohara *et al.* in 2013 (10) managed to regenerate whole pulp with the combination of G-CSF and DPSCs. When compared with the control, it was observed that the combination of both achieved the highest regenerative potential (9,10).

Similar results were observed by Murakami *et al.* (9) who also used G-CSF and DPSCs achieving complete pulp regeneration with high rate of angiogenesis and neurogenesis. In 2016, Iohara *et al.* (11) conducted a new clinical trial in dogs where they used the same technique used in 2013 by this same author (10) who this time treated teeth with pulpal diagnosis of irreversible pulpitis. Unlike the studies mentioned above (6,7,9,10) the evaluation of the animals was by magnetic resonance. The results of this study were also compared with histological methods and sensitivity test. The images made it possible to show that after 180 days there was regeneration of a more radiolucent dentinal tissue, which suggests that there is less mineralization of the dentin formed with the transplant of DPSCs (11). Mangione *et al.* (12)In 2017 they performed a Split Mouth randomized study in minipigs where, when comparing intervened and non-intervened teeth, pulp regeneration was not found when grafting DPSCs, an osteodentine with different characteristics was formed to a normal dentine.

 In the study by Iohara *et al.* in 2011 (7) they observed dentinal regeneration after transplantation of DPSCs. Therefore, they conclude that the DPSCs could allow the regeneration of the entire dentin-pulp complex.

Kodonas *et al.* in 2012 (13) performed a clinical trial in mini pigs with DPSCs. The histological results showed dentinal tissue formation in the canal walls, and the presence of Dentin Matrix Protein-1 was evidenced (DMP-1) and Bone sialoprotein II (BSP-II) which indicate odontoblastic activity which would be responsible for this dentin formation.

Iohara *et al.* in 2004 (14) conducted a clinical trial with dogs and DPSCS. A greater degree of dentin formation could be observed when using pellets of mesenchymal stem cells treated with BMP-2. Gronthos *et al.* in 2000 (15) as in 2002 (16) had already tested dentinal regeneration when using humans but in immunocompromised mice and performing transplantation of xenogenic type. It was in the investigation of Iohara *et al.* in 2004 (14) that dentin was regenerated with autologous transplantation and in healthy animals without the requirement of immunocompromise or immunosuppression. By joining the MSCs with a pellet with BMP-2 *in vitro*, the extracellular matrix of the pellet can be used as a scaffold to manipulate the growth of cells in odontoblasts prior to transplantation. By establishing and optimizing this technique, a treatment with relevance for endodontic and cavities treatment could be achieved (14).

Studies in humans have also been conducted to achieve regeneration of the dentin tissue. In 2011, Lovelace *et al.* (17) conducted a clinical trial in which they performed a revascularization treatment in 8 patients with immature teeth and apical periodontitis. Both imaging and histology showed the apical closure of these immature teeth. This apical closure is explained by the presence of mesenchymal stem cells that reach the interior of the canal through apical bleeding. It is thought that said cells are SCAPs carried by the blood. However, a histological study conducted in 2010 by Wang *et al.* (18) showed that after a revascularization treatment in immature teeth diagnosed with pulpal necrosis or apical periodontitis, an apical closure was achieved but the root canals of the tooth were filled with ectopic bone tissue, fibrous tissue and cement apposition (11,18). More recent studies such as that of Nakashima *et al.* in 2017 (19) carried out a pilot study where they transplanted DPSCs obtained from teeth with irreversible pulpitis. Clinically, histologically and imaging, regeneration of the pulp and dentin could be seen with a sensitivity and vitality of the pulp almost normal in pulpectomized teeth.

-Regeneration of the alveolar bone

48% of the articles included in this review studied bone regeneration. Multiple authors have used DPSCs to regenerate alveolar bone. Ito *et al.* in 2011 (20) conducted an *in vivo* clinical trial in which they used DPSCs. The analysis of the results in radiographic, histological and clinical form showed good bone regeneration using the MSCs, observing a greater amount of bone compared to the control. Regarding the dental implant (IOI), although in both cases they were osseointegrated, it was observed that in the area where the mensenchymal stem cells were used there was more bone around the implant compared to the control.

Yamada *et al.* in 2011 (21) conducted a clinical trial in animals, in which they also obtained bone regeneration after the transplant of DPSCs in the bone defect after a tooth extraction. Yamada *et al.* in 2010 (22) performed a study similar to the previous one in which they tested the capacity of this regenerated bone for the osseointegration of implants. The results obtained showed correct osseointegration when using the different types of mesenchymal stem cells.

Monti *et al.* in 2016 (23) conducted a clinical trial in humans with 6 patients. The alveoli after the extraction were filled with a collagen sponge and DPSCs, other defects were only filled with the collagen sponge as a control. After imaging and clinical analysis, it was observed that the regenerated alveolar bone where DPSCs were implanted was more mature and of higher quality than in the control group after 60 days had elapsed. In addition, the bone regenerated with cells was apt to be rehabilitated based on IOI. However, in the study by Barbier *et al.* in 2018 (24) filled out post-extraction alveoli with DPSCs and a collagen scaffold and did not find a greater bone regeneration of the defect due to impacted third molar extraction when using DPSCs.

Mesenchymal stem cells of the periosteum have been able to regenerate alveolar bone. Mouraret *et al.* in 2014 (25) conducted a clinical trial in animals where they created a vertical defect in mice at the palatal level which was filled with a collagen sponge with periosteum. It was compared to a tunneling technique without the use of a scaffold that was a collagen sponge. When the collagen sponge was not used, bone resorption was observed. Therefore, it was demonstrated that the periosteum has mesenchymal stem cells that participate in bone regeneration, but with the help of a scaffold such as collagen, for example.

Shiehzadeh *et al.* 2014 (26) reported three cases in which they sought to repair bone defects of periapical lesions in teeth with apical peridontitis. For this, in three different patients, they carried out protocols with different mesenchymal stem cells. The first case used SCAPs, the second case used SHEDs and the third case used cells obtained with a new method in this study that obtained cells from the periapical tissues through the canal (iPAPs). With a radiographic method it was possible to see the repair of the lesions together with the apical closure of the immature teeth, clinically there were no complications or symptomatology after the treatment.

Chen *et al.* in 2016 (27) conducted a randomized clinical trial in humans, where they used mesenchymal stem cells of the periodontal ligament (PLSCs). In 30 patients, 41 teeth were intervened in total with periodontal defects, which were treated with PLSCs obtained from previously extracted third molars. Although radiological and clinically greater regeneration was seen when using MSC, the difference was not statistically significant after 12 months.

Khojasteh *et al.* in 2016 (28) conducted a human clinical trial this time using cells from the adipose body of the cheek (ASCs). The clinical, histological and radiographic evaluation of the patients showed that combining the scaffold with the mesenchymal stem cells obtained better bone regeneration with good volume and preventing postoperative bone resorption (28).

Redondo *et al.* in 2018 (29) conducted a pilot study where they obtained BMSCs of maxillary bone and managed to regenerate the alveolar bone in cavities of maxillary radicular cysts when using scaffolding of Serum cross liked scaffold.

-Regeneration of the periodontium

One study (5,8%) described the regeneration of periodontal tissues in animals. In the clinical trial of Nakajima *et al.* (30) conducted in 2014, mesenchymal stem cells were extracted from the granulation tissue (TGSCs) generated in alveoli after performing premolar extraction. The regeneration obtained was analyzed histologically, which resulted in a periodontium with periodontal ligament, cement and bone. The origin of these cells still uncertain, being likely to come from remains of the periodontal ligament, the apical papilla, the alveolar bone or surrounding MSCs. This is why the granulation tissue cannot yet be determined as a proper niche of stem cells.

Studies have been found with allogenic mesenchymal stem cells that have demonstrated the ability of mesenchymal stem cells to regenerate the periodontium.

-Comparison with extraoral mesenchymal stem cells

In 2015, Murakami *et al.* (9) compared the pulp mesenchymal stem cells with those of the bone marrow of the sternum. The protocol carried out was the same, only that the origin of the mesenchymal cell used changed. The results showed that in order to regenerate the dental pulp, the bone marrow cells obtained a greater quantity of pulp matrix, but the cells of the dental pulp managed to regenerate greater vascularization and innervation.

Yamada *et al.* in 2004 (31) studied the use of mesenchymal stem cells of the bone marrow in order to regenerate alveolar bone for implants, thus regenerating a functional bone for it. Then Yamada *et al.* in their 2011 studies (21) and 2010 (22) also compared DPSCs and SHEDs with the BMSCs of the iliac crest but this time in order to regenerate bone alveolar. The results showed a good bone regeneration by the three types of cells, and bone apt to rehabilitate with implants. Therefore, DPSCs and SHEDs could be an alternative to regenerate bone, obtaining the same results as with BMSCs. In the study by Ito *et al.* in 2011 (20) they also compared the DPSCs with the BMSCs to regenerate the alveolar bone, they observed greater osteogenic potential from the DPSCs, although both cell types regenerated bone suiTable for osseointegrating implants.

Finally, there are intraoral studies with extra oral mesenchymal stem cells specifically of adipose tissue of the abdomen. In the study by Murakami *et al.* 2015 (9) they compared the DPSCs with the ASCs of the abdomen. The results were the same as with the BMSCs, that is, obtaining pulp matrix but with less vascularization and innervation compared to that obtained with DPSCs. Iohara *et al.* in their 2011 study (7) compared DPSCs with ASCs in order to regenerate dental pulp and dentin for apical closure. The histological results showed a low potential of the fat cells to regenerate the dentin-pulp complex.

In conclusion, although most of the studies were performed *in vivo*, it can be noted that PLSCs, DPSCs, ASCs, SCAPs, SHEDs were able to regenerate alveolar bone, DPSCs and SCAPs the dentin-pulp complex and the TGSCs the periodontium. It should be noted that the studies used in this review are not of high quality, so there is a need in the literature to conduct more randomized controlled clinical trials with a larger sample size and homogeneity in follow-up times so that the results obtained are truly significant. and extrapolated to clinical use. It is necessary to mention that not only the cells influence the results, so the use of different scaffolds and biomolecules in the environments in the different studies means that these are not really comparable. This is why it is necessary to standardize the studies with the same scaffolds and biomolecules in order to effectively compare the results obtained using mesenchymal stem cells from different areas in the same environment. Although to date it can be concluded that the results obtained when regenerating using mesenchymal stem cells are positive, it is necessary to determine the best scaffolding and the best means to obtain the best results.
